# Younger age and female gender are determinants of underestimated cardiovascular risk in rheumatoid arthritis patients: a prospective cohort study

**DOI:** 10.1186/s13075-020-02384-9

**Published:** 2021-01-04

**Authors:** Daphne C. Rohrich, Eline H. M. van de Wetering, Alexander J. Rennings, Elke E. Arts, Inger L. Meek, Alfons A. den Broeder, Jaap Fransen, Calin D. Popa

**Affiliations:** 1 Department of Rheumatology, Sint Maartenskliniek, Hengstdal nr. 3, 6574 NA Ubbergen, The Netherlands; 2 Department of Rheumatology, Radboud University Nijmegen Medical Centre, Geert Grooteplein 8, 6500 HB Nijmegen, The Netherlands; 3 Department of Internal Medicine, Radboud University Nijmegen Medical Centre, Geert Grooteplein 8, 6500 HB Nijmegen, The Netherlands

**Keywords:** Rheumatoid arthritis, Cardiovascular, Risk factors, Age, Gender

## Abstract

**Background:**

Rheumatoid arthritis (RA) patients have an increased cardiovascular (CV) risk. Here, we aimed to investigate whether gender and age are contributing to the misclassification of CV risk in RA patients.

**Methods:**

Prospectively collected data on cardiovascular risk factors and incident events from the Nijmegen inception cohort were analyzed, with up to 10 years follow-up. Original as well as the EULAR-modified (M)_SCORE algorithms were used to calculate CV risk. Patients were stratified in deciles according to predicted risk; the Hosmer-Lemeshow test was used to check concordance between observed and predicted risk, in subgroups of gender and age.

**Results:**

There were 863 RA patients included with 128 incident CV events. When using SCORE in the whole group, there was evidence of a discrepancy between the predicted and observed CV risk (H-L test *p* < 0.003), mainly present in the female subgroup (H-L test *p* < 0.001). Interestingly, 36% of females who developed an event belonged to the low CV risk group, whereas this was just 10% in RA males. When analyzing the subgroups based on age, a discrepancy was present only in the youngest patients (H-L test *p* < 0.001 in patients < 55 years) consisting of an underestimation of CV risk (5.3% predicted vs. 8.0% observed). Similar results were obtained when the M_SCORE was applied.

**Conclusion:**

CV risk is especially underestimated in female and younger RA patients. This suggests that modifying the weight for the female gender and/or younger age in currently used CV risk algorithms might improve their predictive value in RA, contributing to better CV risk management.

## Introduction

Rheumatoid arthritis (RA) patients have a higher risk of developing cardiovascular diseases (CVD) than the general population [1]. It has been previously suggested that this increased risk of CVD is in part due to systemic inflammation seen in patients with RA, although excess in classical risk factors like smoking and obesity also play a role [2].

Current algorithms developed to predict CVD, such as the Systematic Coronary Risk Evaluation score (SCORE), underestimate the risk in RA patients, especially in those patients originally classified as having low or intermediate risk [3]. It is well known that older age leads to higher CV risk in the general population. Likewise, males are at greater risk compared to females. However, the results of a recent meta-analysis suggested that compared to the general population, younger RA patients bear the greatest relative risk of developing CV events, whereas older RA patients seem to have similar relative risks when compared to age-matched counterparts [4]. Furthermore, women with RA seem to be at a greater CV risk than those without RA [5]. Early menopause seems to be a predictor for RA, and in addition, early menopause in women with RA may lead to a higher CVD risk [6–8]. Therefore, one can suggest that gender and age are likely to have a distinct impact on CVD risk in RA patients than in the general population.

Because the majority of risk calculators such as the SCORE risk charts use standard weighing of gender and age, their unadjusted use to patients with RA would yield inappropriate estimates of the CV risk. Because this hypothesis has been generated from results coming from a meta-analysis, we aimed in this study to confirm in a prospective follow-up inception cohort of RA patients whether age and gender are contributing to the underestimation, and thus misclassification, of CV risk in RA when current risk algorithms developed for the general population are used. Adjusting the impact of these parameters to better suit the RA population has not been part of the present investigation.

## Patients and methods

### Design

For this study, prospectively collected data on cardiovascular risk factors and incident events from the Nijmegen early RA inception cohort, with a follow-up of up to 10 years, were used. In the cohort, patients had been included at diagnosis of RA (baseline) in the outpatient clinic of the Department of Rheumatology of the Radboud University Nijmegen Medical Centre (since 1985) or the Maartenskliniek Nijmegen (since 1990). Patients were included when they had a disease duration of < 1 year, were disease-modifying antirheumatic drug (DMARD) naive, and fulfilled the 1987 American College of Rheumatology (ACR) criteria (before 2010) or the ACR/EULAR 2010 criteria. All patients provided written informed consent.

For the current analysis, patients with a history of CVD prior to RA diagnosis were excluded. The SCORE and the EULAR-modified SCORE algorithm were used for the prediction of CVD risk [9].

### Baseline data

Baseline characteristics were retrieved from the cohort database, including age (years), gender (male/female), rheumatoid factor (RF) positivity, anti-cyclic citrullinated peptide (aCCP) antibody positivity, and Disease Activity Score (DAS-28) at disease diagnosis. Baseline data regarding CV risk factors were collected by medical chart and electronic patient file review, including smoking status (Y/N), blood pressure (mmHg), height (m), weight (kg), diabetes mellitus (Y/N), hypertension (Y/N), and family history of CVD (Y/N). Non-fasting total cholesterol and high-density lipoprotein cholesterol concentrations (mmol/l) were measured according to the standard laboratory procedures. CV events within 10 years were retrieved from physician diagnosis and extensive review of medical charts and electronic patient files. Acute coronary syndrome (myocardial infarction and unstable angina pectoris), stable angina pectoris (sAP), cerebral vascular accident (CVA), transient ischemic attack (TIA), peripheral artery disease (PAD), percutaneous transluminal coronary angioplasty (PTCA), coronary artery bypass grafting (CABG), and coronary angiography (CAG) were included CV events. Deaths due to CVD were verified from death certificates, provided by the Statistics Netherlands [10], including deaths due to CVD and CVA but excluding cerebral hemorrhage and non-coronary cardiac death.

### Statistical analysis

Baseline data were used to calculate individual risks for CV events within 10 years for both CV risk algorithms. Missing values were imputed using multiple imputations with five repetitions. Baseline differences between the groups of RA patients with or without a CV event at follow-up were analyzed using the *t* test or *χ*^2^ test, as appropriate. The predicted risks for a CV event in patients with a follow-up time of < 10 years were adjusted proportionally, according to the length of actual follow-up, and calculated as a proportion of 10 years. The Hosmer-Lemeshow (H-L) test was used to check the concordance between the observed and predicted risk, in subgroups based on gender and age. All statistical analyses were performed using SPSS V.20.0.

## Results

### Patient characteristics

A total of 863 prospectively followed RA patients were included in the analysis, of whom 566 were female and 297 were male. During the follow-up, 128 cardiovascular events had been recorded. Expectedly, patients who developed CV events had more classical CV risk factors at baseline, such as high blood pressure or smoking (Table 1). On average, patients with CVD events were older and were more likely to be male compared to patients without CVD events. The DAS-28 was higher in patients with CVD, but there were no differences in the occurrence of rheumatoid factor (RF) or anti-CCP antibodies between the two groups (Table 1).

**Table 1:** Patient characteristics in the two groups, with and without incident CV events

Parameter	CVD− (***N*** = 735)	CVD+ (***N*** = 128)	***p*** value
Age (years)	53.2 ± 13.6	61.2 ± 10.2	< 0.0001
Gender, F (%)	68	52	< 0.0001
Smoker (%)	30	41	0.042
TC (mmol/l)	5.22 ± 1.23	5.26 ± 1.34	0.72
HDL (mmol/l)	1.30 ± 0.30	1.24 ± 0.28	0.040
TC:HDL	4.11 ± 0.87	4.32 ± 0.96	0.014
SBP (mmHg)	146 ± 23	155 ± 24	< 0.0001
DBP (mmHg)	84 ± 12	86 ± 9	0.038
DAS-28	4.84 ± 1.26	5.39 ± 1.33	< 0.0001
RF+ (%)	74	79	0.27
*aCCP+ (%)*	*67*	*66*	*0.83*

Results are expressed as percentages or as means ± standard deviation (SD)

*CVD* cardiovascular disease, *F* female, *TC* total cholesterol, *HDL* high-density lipoprotein, *SBP* systolic blood pressure, *DBP* diastolic blood pressure, *DAS-28* Disease Activity Score, *RF* rheumatoid factor, *aCCP* anti-cyclic citrullinated peptide

### Influence of gender on CVD risk prediction

**Fig. 1:**
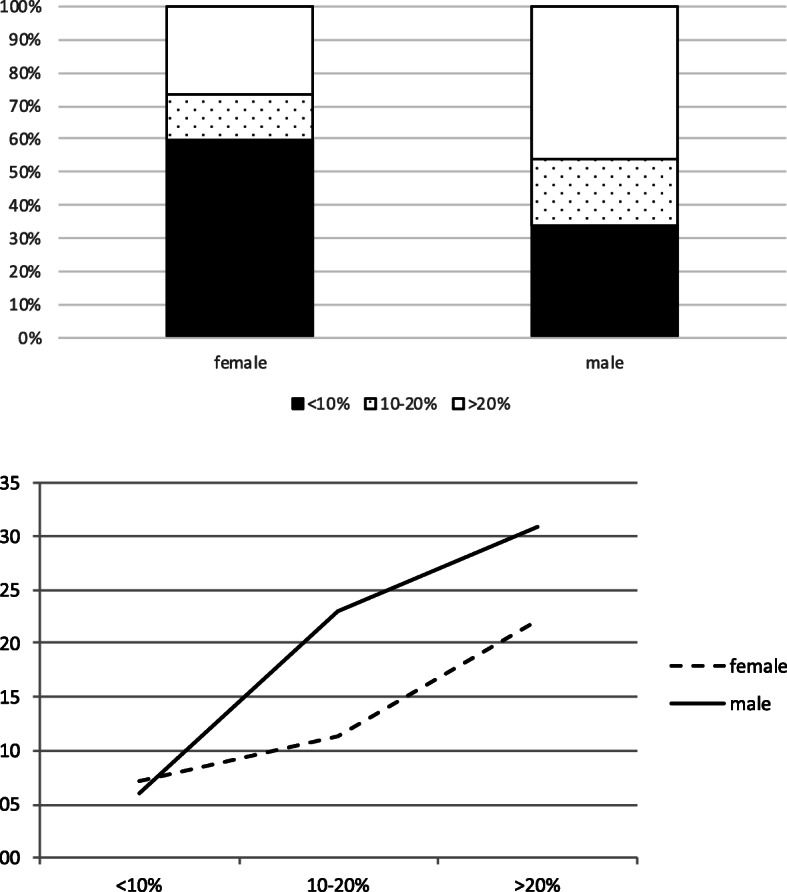
The distribution of CV risk. **a** The distribution of CV risk among women and men with RA: low CV risk (white bar), intermediary CV risk (gray bar), and high CV risk (black bar). **b** The observed percentages (*Y*-axis) of female (dotted line) and male (black line) RA patients who experienced a new CV event during the follow-up period, according to their predicted CV risk category (*X*-axis): low (< 10%), intermediary (10–20%), and high (> 20%)

As shown (Fig. 1a), in the group of females with RA, most patients had a low predicted CV risk (59%), whereas in RA males, 68% had an intermediary or high (46%) CV risk. In the total group, there was a discrepancy between predicted and observed CV risk (H-L test *p* < 0.003) when the SCORE algorithm was applied. When analyzed separately for females and males (Fig. 1b), this discrepancy appeared to be especially present in the female subgroup (H-L test *p* < 0.001), rather than in the male subgroup (H-L test *p* = 0.09). Interestingly, in the group of RA patients having a low predicted CV risk (< 10%), the proportion of females still experiencing an incident CV event at follow-up is higher than that of the males and higher than predicted as well (Table 2). This was not the case in the other two risk groups (intermediary and high CV risk group), where males had a CV event more often (Table 2).

**Table 2:** Observed CV event distribution in the studied group, according to gender and CV risk group

CV risk calculator	< 10%			10–20%			> 20%		
	*N*	CVD+	%(AR)	*N*	CVD+	%(AR)	*N*	CVD+	%(AR)
**SCORE**									
F	336	24	7.1	80	9	11.3	149	33	22.1
M	100	6	6.0	61	14	23.0	136	42	30.9
T	436	30	6.9	141	23	16.3	285	75	26.3
**FRS**									
F	271	13	4.8	168	23	13.7	127	30	23.6
M	65	2	3.1	77	14	18.2	155	47	30.3
T	336	15	4.5	245	37	15.1	282	77	27.3
**QR2**									
F	262	14	5.3	129	17	13.2	175	35	20.0
M	68	4	5.9	69	9	13.0	160	50	31.1
T	330	18	5.5	198	26	13.1	335	85	25.4

*CV* cardiovascular, *CVD* cardiovascular disease, *AR* absolute risk, *FRS* Framingham Risk Score, *QR2* QRisk2, *F* females, *M* males, *T* total

**Fig. 2:**
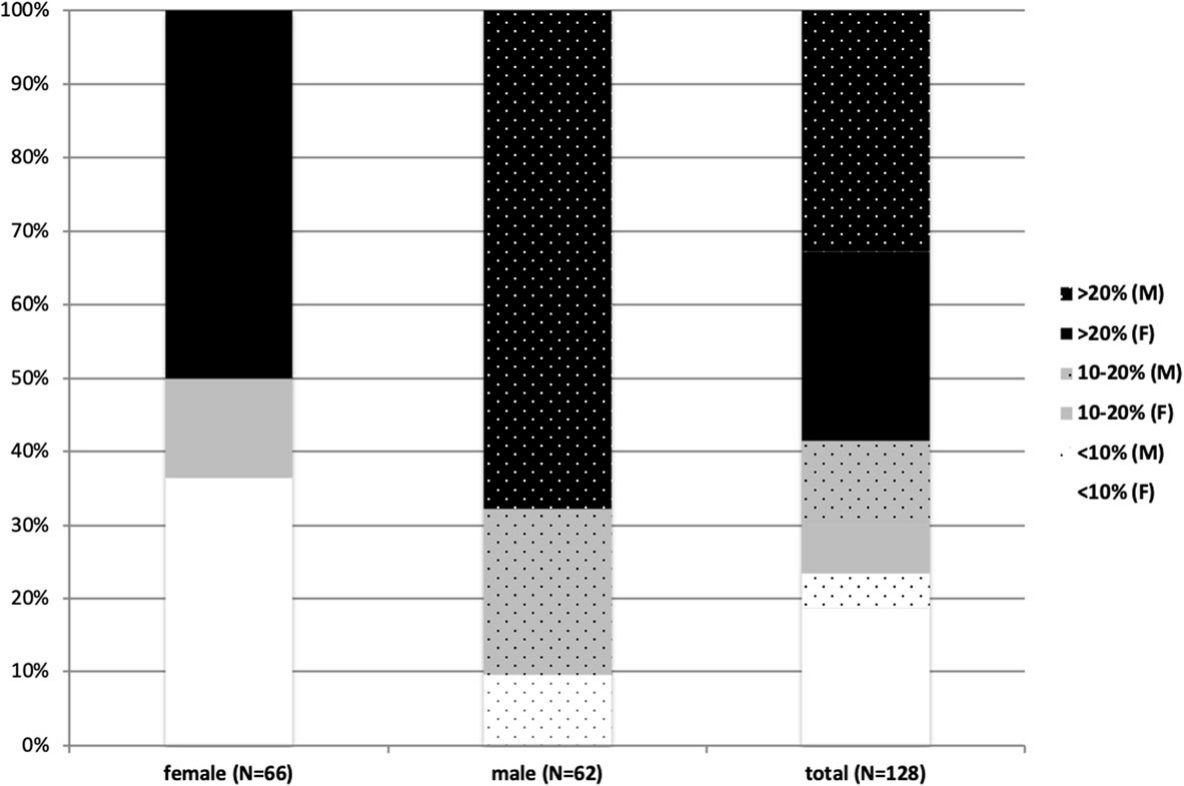
Rheumatoid arthritis patients and CV events. The distribution in percentages of RA patients who experienced a CV event according to their gender and their initially assigned CV risk group

When looking at the distribution of RA patients who experienced a CV event by gender and their initially assigned CV risk group (Fig. 2), it appeared that among the group of females who developed CVD, 36% had a predicted risk lower than 10% (low predicted risk). In comparison, only 10% of males who developed CVD had a predicted risk lower than 10% (Fig. 2). Overall, the majority of RA patients who developed CVD and have been initially assigned to the lower-risk group were eventually women, whereas RA men were more often seen in the intermediary- and high-risk groups (Fig. 2). Similar results were obtained when the M-SCORE was applied.

### Influence of age on CVD risk prediction

**Fig. 3:**
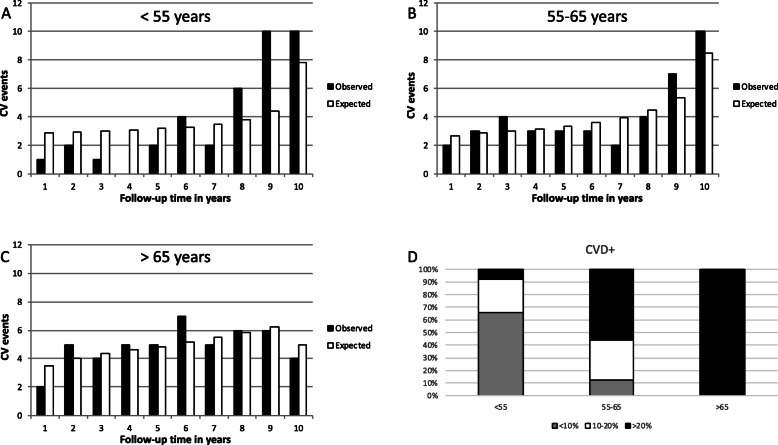
CV risk according to age group. The differences between the predicted (white bars) and observed (black bars) CV risk in RA patients younger than 55 years (**a**), between 55 and 65 years (**b**), and older than 65 years (**c**). The distribution in percentages of RA patients who experienced a CV event according to their age and their initially assigned CV risk group (**d**)

To analyze the influence of age on CVD risk prediction, subgroups were made based on age: < 55 years, 55–65 years, and > 65 years. Almost 30% of all the CV events had been registered in the youngest RA patients’ group (< 55 years), whereas the oldest patients (> 65 years) accounted for 38% of all CV events (Table 3). The H-L test values of the three age groups were *p* < 0.001, *p* = 0.93, and *p* = 0.96, respectively (Fig. 3), pointing to the misclassification (underestimation) of CV risk in the youngest RA patients (5.3% predicted vs. 8.0% observed). Similar results were obtained when the M-SCORE was applied.

**Table 3:** CV event distribution in the studied group, according to age and CV risk group

Age (years)	SCORE risk group < 10%			SCORE risk group 10–20%			SCORE risk group > 20%			Total CVD+
	*N*	CVD+	%(AR)	*N*	CVD+	%(AR)	*N*	CVD+	%(AR)	
< 55	381	25	7	41	10	24	16	3	19	38
55–65	55	5	9	89	13	15	95	23	24	41
> 65	0	0	–	11	0	0	174	49	28	49

*CVD* cardiovascular disease, *AR* absolute risk

## Discussion

According to the results of this study, younger (< 55) RA patients and females with RA are likely to form two subgroups with the largest underestimation of CVD risk, if CV risk algorithms developed for the general population (SCORE) were used. The present results confirm those previously obtained in meta-analysis [4]. RA is associated with an increased risk of developing acute CV events. Following EULAR recommendations [9], patients receiving the diagnosis RA should be further monitored for the presence of risk factors for CVD. Risk management should be initiated by firstly determining the 10-year risk to develop CV events. Various risk calculators have been used so far, yet none of them appears to perform in RA patients as well as in the general population [3]. Attempts to modify these algorithms in order to better fit the RA population, including the addition of inflammatory parameters, disease activity markers, or even genetic markers, have yielded disappointing results [11–13], although one recent algorithm might lead to some improvement in accuracy [14]. The results of the present study yield the hypothesis of modifying the weight of gender and/or age in current CV risk calculators to better fit RA patients. This hypothesis warrants further attention in the future, yet it does not constitute the aim of the present study.

In the current study, RA females accounted for the vast majority of CV events in the low-risk group (80%), which eventually represented over one third of all CV events in RA women. In comparison, the distribution of CV events in RA men was more in accordance with their predicted risk category, being the lowest (10%) in the low-risk group and highest (68%) in RA men initially assigned to the high-risk group. It seems therefore that at diagnosis, CVD risk is especially underestimated in females, and there are indeed several putative explanations for this. Firstly, previous studies have suggested that females and males with RA are not equally affected by inflammation with respect to CV risk factors. In line with this, we have previously shown that compared to healthy volunteers, the HDL-2 subfraction is declined in females with RA but not in males [5]. Also, it is hypothesized that due to systemic inflammation, females with RA reach menopause earlier than normally expected, which is augmenting their CV risk as compared to non-RA women of similar age [6, 15]. Finally, low-grade inflammation and a disturbed metabolism, as it is the case in diabetes mellitus, may augment the CV risk much more in women (relative risk 3 to 8 times higher) than in men (relative risk 2 to 3 times higher) as compared to the general population [16].

In our study, CV events occurred very often even in young RA patients and accounted eventually for almost 30% of all the registered CV events. Among them, two thirds have been initially assigned to the group of low CV risk at baseline. Age is a very strong predictor of future CV risk. According to the SCORE chart in The Netherlands, women under 55 and men under 50 would nearly always have a low CV risk, independent of the other CV risk factors such as dyslipidemia, hypertension, and smoking status. Consequently, according to the epidemiological data, individuals belonging to this risk category should not initiate drug therapies, as these would have a very limited impact on their CV risk, which is low already. Nevertheless, previous studies performed in RA populations have indicated the presence of atherosclerotic plaques even in younger patients and/or patients assigned to the low CV risk category [17, 18]. Accordingly, one third of RA women who had a low SCORE value and are aged > 49.5 years or/and have a total cholesterol concentration of > 5.4 mmol/l experience high-risk atherosclerosis and would therefore require intensive CVD risk management [19]. As carotid artery intima-media thickness (cIMT) and coronary artery calcification (CAC) score are both surrogate markers of underlying atherosclerosis and are both associated with increased CV risk, this suggests that these patients bear a higher CV risk than the one initially assigned using just the SCORE risk calculator. Of note, cIMT is likely to be more sensitive than CAC in RA patients in order to detect subclinical atherosclerosis associated with high CV risk [20]. Our results strengthen this hypothesis from an epidemiological perspective, as the number of the observed CV events was higher than predicted in the low-CV risk group of patients. These observations might also be explained from a pathophysiological perspective. Atherosclerosis is accelerated in RA patients [21], most probably due to dyslipidemia, which is widely present [5, 22], and due to inflammation during periods of active disease, which may contribute to plaque development and instability/rupture [23]. In other words, it seems that RA patients would need less time (thus would be younger) to reach a critical level of vulnerability of atherosclerotic lesions that would trigger an acute event, as compared to the general population, as suggested by the data of the present study and our previous meta-analysis [4]. We are aware of the low absolute risk in the lower CV risk category patients. Nevertheless, we consider that these results cannot be neglected and should lead to a better performance of risk predictions in the “low-risk” group. Because this is the group where most health gain may be achieved, as these patients have the highest chance of being most often undertreated for their traditional CV risk factors and therefore more prone to develop CVD.

Our study has a few limitations. Firstly, the number of patients included is limited as compared with studies of CVD in the general population. Yet, the cohort used is well and prospectively documented, and also one of the largest single-center cohorts of its kind. Secondly, most of the patients investigated were included before CVD risk management had been implemented, limiting the use of absolute risk predictions. Thirdly, our study investigated if current algorithms have a proper calibration in the subgroups tested (i.e., gender and age-specific subgroups). No statistical comparisons of calibrations between the groups have been made. Finally, data on the use of non-steroidal anti-inflammatory drugs (NSAIDs) and corticosteroids are missing. This might have influenced the results of the study, though some recent reports suggest that the use of NSAIDs does not increase CV incidence in the RA population as compared to osteoarthritis (OA) [24].

## Conclusions

In conclusion, the results of our study suggest that the incidence of CVD among women and young RA patients initially assigned to the low-risk category is higher than predicted using current algorithms. Consequently, the CV risk in these subgroups seems underestimated. Whether modifying the weight for the female gender and/or younger age in the risk algorithms would result in better CV risk predictions in RA remains a subject to be investigated in future studies. Alternatively, other strategies (e.g., biomarkers, cIMT, or CAC measurements) aiming at the same goal could be envisaged in order to improve CV risk management in patients with RA and/or other chronic inflammatory conditions.

## Data Availability

The datasets used and/or analyzed during the current study are available from the corresponding author on reasonable request.

## Abbreviations

RA: Rheumatoid arthritis
CV: Cardiovascular
EULAR: European League Against Rheumatism
H-L test: Hosmer-Lemeshow test
CVD: Cardiovascular diseases
SCORE score: Systematic Coronary Risk Evaluation score
DMARD: Disease-modifying antirheumatic drug
ACR: American College of Rheumatology
RF: Rheumatoid factor
aCCP: Anti-cyclic citrullinated peptide
DAS-28: Disease Activity Score
sAP: Stable angina pectoris
CVA: Cerebral vascular accident
TIA: Transient ischemic attack
PAD: Peripheral artery disease
PTCA: Percutaneous transluminal coronary angioplasty
CABG: Coronary artery bypass grafting
CAG: Coronary angiography
TC: Total cholesterol
HDL: High-density lipoprotein
SBP: Systolic blood pressure
DBP: Diastolic blood pressure
cIMT: Carotid artery intima-media thickness
CAC: Coronary artery calcification
NSAIDs: Non-steroidal anti-inflammatory drugs
OA: Osteoarthritis
